# A Novel Bio-Purification Process Employing an Engineered *E. coli* Strain for Downstream Processing of Lactic Acid Solutions from the Fermentation of Agro-Industrial by-Products

**DOI:** 10.3390/bioengineering11050412

**Published:** 2024-04-23

**Authors:** Alexandra Nastouli, Alexandra Moschona, Panagiotis A. Bizirtsakis, Joseph Sweeney, Irini Angelidaki, Michael Harasek, Anastasios J. Karabelas, Sotiris I. Patsios

**Affiliations:** 1 Laboratory of Natural Resources and Renewable Energies, Chemical Process & Energy Resources Institute (CPERI), Centre for Research and Technology-Hellas (CERTH), GR 57001 Thessaloniki, Greece; a.nastouli@certh.gr (A.N.); alexmoschona@certh.gr (A.M.); pbizirtsakis@certh.gr (P.A.B.); karabaj@certh.gr (A.J.K.); 2 Institute of Chemical, Environmental and Bioscience Engineering, TU Wien, AT 1060 Vienna, Austria; michael.harasek@tuwien.ac.at; 3 School of Biosystems and Food Engineering, University College Dublin (UCD), D04 V1W8 Dublin, Ireland; joseph.sweeney@ucd.ie; 4 Department of Chemical and Biochemical Engineering, Technical University of Denmark, DK-2800 Kongens Lyngby, Denmark; iria@kt.dtu.dk

**Keywords:** bio-purification, lactic acid, membrane bioreactor, ultrafiltration, nanofiltration, *E. coli*, fermentation, semi-continuous operation, selective separation

## Abstract

This study aims to integrate a novel bio-purification process employing an engineered *E. coli* strain in the downstream processing of lactic acid (LA) fermentation broths from low-cost renewable biological feedstocks. Fermentation broth of candy waste and digestate mixture was used as a real biological feedstock. An engineered *E. coli* strain that selectively catabolize impurities without catabolizing LA was initially adapted on the biological feedstock, followed by shake flask experiments to prove the bio-purification concept. Scale-up and validation in a bench-scale bioreactor followed, before developing a semi-continuous membrane bioreactor (MBR) bio-purification process. The MBR bio-purification was assessed with biological feedstocks which simulated ultrafiltration or nanofiltration permeates. Incomplete removal of impurities and increased fouling was observed in the case of the ultrafiltration permeate. Contrarily, the nanofiltration permeate was successfully treated with MBR bio-purification, since low membrane fouling, 100% maltose and acetic acid removal, and no LA catabolism was achieved. MBR bio-purification as a post-treatment step in the downstream processing of LA was demonstrated as a promising technology for increasing the purity of LA solutions.

## 1. Introduction

Natural resource overexploitation together with the increase in global population and waste production necessitates the transition from a linear to a circular bio-based economy. Alternative technological solutions that are put forth aiming at the development of a bio-based economy include biorefineries and novel waste-to-energy and waste-to-bioproducts processes. However, a significant challenge is that, in many cases, feedstocks used in biorefineries compete with food [1,2]. For instance, 1st generation feedstocks (such as sugarcane, corn starch, sugar beet, etc.), which are currently used for the fermentative production of lactic acid (LA) at an industrial level [3,4], can be also used for food and feed applications. Therefore, alternative and inexpensive feedstocks, such as residual biomasses and agro-industrial by-products/wastes are gaining increasing attention for the production of value-added products. Many agricultural residues and food wastes, which are commonly treated through anaerobic digestion, may be used as feedstock for LA microbial fermentation due to their high sugar content [3]. Researchers have studied LA production from different waste mixtures such as a mixture of candy waste and digestate or a mixture of municipal organic waste, supermarket waste, and food waste (i.e., bio-pulp) [5,6,7].

LA is a platform chemical with 2 stereoisomers D (−) and L (+), in which approx. 90% of its total demand is produced biochemically (lactic acid fermentation) [8,9]. It is widely used industrially for food and feed applications, as a bio-chemical, and for bio-polymer production. In particular, green transition and environmental awareness have increased poly-lactic acid (PLA) world demand due to its biodegradability [9,10]. Except for the availability and cost of the feedstocks used for LA production, downstream processing remains another challenge due to the characteristics of the fermentation broth [11,12,13]. Specific impurities contained in the LA fermentation broth, such as organic acids, have high physicochemical affinity with LA that renders purification through conventional physicochemical methods quite challenging. In addition, LA is a thermally unstable molecule with strong H_2_O affinity [14].

**Figure 1:**
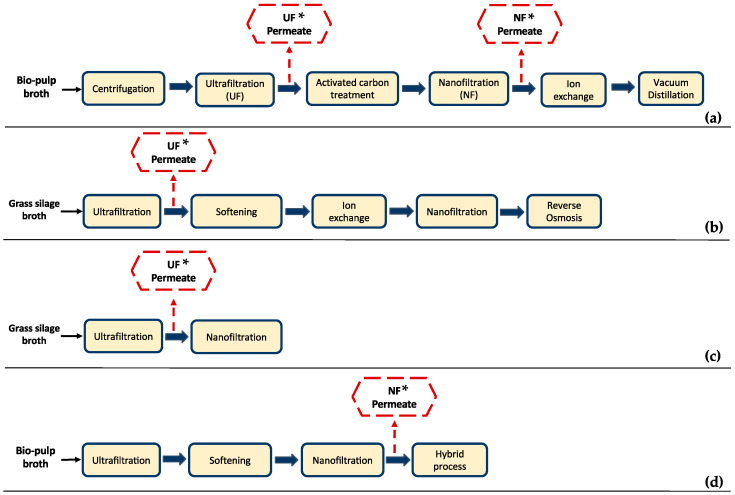
Examples of downstream processing systems of lactic acid (LA) fermentation broths employing membrane technologies; the typical composition of the permeate streams marked with an asterisk (*) are summarized in Table 1. (**a**) Alvarado-Morales et al. [6]; (**b**) Schaffenberger [19]; (**c**) J. Ecker et al. [17]; (**d**) Alvarado-Morales et al. [6].

Many researchers have focused on membrane separation technologies for the recovery and purification of LA from the fermentation broth (Figure 1). An integrated hybrid system is usually suggested, where different separation technologies are combined to achieve high LA purity. Microfiltration (MF), ultrafiltration (UF), and nanofiltration (NF) are applied in the majority of these studies as part of the downstream processing [6,15,16,17,18,19]. MF and/or UF are mainly applied for the removal of cells, suspended solids (e.g., cell debris), and/or organic macromolecules (e.g., polysaccharides, proteins, etc.), whereas NF can be used for the removal of soluble compounds and/or product concentrations.

For instance, Alvarado-Morales et al. [6] studied the recovery of LA from bio-pulp fermentation broth, which included UF and activated carbon treatment as pre-purification steps, followed by ion exchange (IE) and vacuum distillation (VD) in the 1st case scenario (Figure 1a). In the 2nd case scenario, an NF step was introduced before the IE process (Figure 1d). Introduction of the NF process increased the purity of LA from 72.50 ± 2.0% to 82.0 ± 1.5% in the 2nd case scenario. Although high LA purity was achieved, impurities such as acetic acid (AA) and formic acid were not totally removed, neither after NF nor IE process steps (Table 1), denoting the limitations of physicochemical technologies for the separation of substances with high chemical affinity to LA.

Schaffenberger [19] studied an alternative to the established industrial process for the production of LA from grass silage juice at the Green Biorefinery (GBR) in Upper Austria. Conventional GBR Upper Austria downstream processing included screw pressing of the grass silage, followed by UF, NF, and electrodialysis (ED) for the recovery of LA, while an alternative process scheme comprised screw pressing, UF, softening, IE, NF, and reverse osmosis (RO) (Figure 1b). IE effluent comprised approx. 15–20 g/L LA, 2–10 g/L AA, and 2–20 g/L sugars. The components of the effluent varied depending on the composition of the IE feed. The study mainly focused on the recovery of amino acids; thus, there are insufficient data available concerning the LA effluent composition in the final process steps; UF permeate composition is indicatively presented in Table 1.

A pilot scale set-up for the recovery of LA and amino acids from grass silage juice was also studied by J Ecker et al. [18], who implemented a strategy that included UF and softening followed by a hybrid process, i.e., a combination of NF, ED, and RO (Figure 1b). Concerning the removal of impurities, a low efficiency was observed as butyric acid and AA were present in the enriched LA solution. Therefore, the need for applying highly selective separation processes to achieve high LA purity (depending on the usage and quality requirements) is obvious. The typical composition of the NF permeate of this study is also presented in Table 1.

J. Ecker et al. [17] studied the optimization of LA and AA separation from grass silage leachate by applying a single NF step, as depicted in Figure 1c. The feedstock was first treated through a UF membrane, and then six different NF membrane modules were tested focusing on the membrane material and process parameters variation. The composition of the UF permeate used in this study is shown in Table 1. The researchers concluded that the investigated NF processes did not achieve a sufficient level of purity; nonetheless, the NF process could be useful as an intermediate step of a downstream processing strategy. Considering the results of the presented literature examples, it can be clearly stated that multiple steps and different separation processes are needed to achieve high LA purity. However, their application at a larger scale is restricted due to high energy and equipment costs [15], resulting in a costly downstream processing [12].

Concerning substances with high physicochemical affinity, a bio-purification process provides a comparative advantage to the conventional physicochemical separation processes [20]. Metabolic engineering advances play an important role behind the bio-purification concept, which is a novel approach for the purification of value-added products. Microorganisms can be used as separation tools since their metabolism can be directed on a target product or a specific activity with very high selectivity. Wang et al. [21] focused on Hg^2+^ bio-detection and elimination by the engineered *E. coli DH5a* strain, while Cheng et al. [22] suggested a strategy for the bio-purification of L-arabinose with yeast. Diaz et al. [23] presented a study focusing on preferential consumption of glycose and xylose by *E. coli* strains for the production of pentose- and hexose-derived products, and Liang et al. [24] established a novel method for the purification of D-tagatose through the selective metabolism of D-galactose by *S. cerevisiae*. Concerning LA, a bio-purification process for PLA was investigated, based on the selective catabolism of D-lactate over L-lactate by an engineered *E. coli* strain (DC1001) [25,26]. However, scale-up of such biotechnological processes is challenging, notably due to the difficulty to achieve efficient separation of the active cells from the treated feedstock/target product and to perform fermentation under a semi-continuous or continuous mode that is advantageous for large-scale applications. The membrane bioreactor (MBR) concept (which is an established technology for wastewater treatment) combining a biotechnological process and membrane separation is quite promising to address these issues.

Recently, Nastouli et al. [20] demonstrated a bio-purification process for synthetic LA solutions, employing an engineered *E. coli* strain cultivated in a MBR. The *E. coli* strain (A1:ldhA) selectively removed impurities, i.e., glucose and AA, from a synthetic medium simulating the composition of grass silage leachate (Table 1). It was shown that for LA concentrations higher than 10 g/L, the growth and activity of the *E. coli* strain was partially hindered. However, at lower LA concentrations, complete removal of glucose and AA (at an initial 0.5 g/L concentration of both impurities) was achieved, while LA concentration remained constant. In addition, a bacteria-free effluent with higher LA purity was obtained under semi-continuous operation of a lab-scale MBR, while the membrane fouling was low during the bench-scale proof-of-concept experiments.

The objective of the present study is to demonstrate the feasibility of applying the aforementioned MBR LA bio-purification process [20] to real fermentation broth representative of a process stream that is obtained after some preliminary downstream processing steps. Considering that the composition of the process streams differs according to the characteristics of the initial feed stream and the downstream processing stage, two specific downstream processing streams, i.e., a permeate after a preliminary UF (or alternatively MF) separation step and a permeate of a subsequent NF separation step, were selected (Table 1). Real LA fermentation broth originating from the fermentation of a mixture of candy waste and digestate was used in this study [20] after either a MF separation step [5] or an NF separation step (this study). A comparative assessment of the performance of the lab-scale MBR set-up for LA bio-purification employing the aforementioned feedstocks (MF and NF permeate) was investigated, providing insights into the potential integration of the proposed LA bio-purification process in the downstream processing of real LA fermentation broths.

## 2. Materials and Methods

### 2.1. Strain and Media

*E. coli* A1:ldhA was provided by a co-author’s (J. B. Sweeney) laboratory, as in our previous study [20]. *E. coli* A1:ldhA is based on strain A1, which is described by Sweeney et al. [27] as consisting of strain W3110—in which the six genes *∆lldD*, *dld*, *glcD*, *ykgF*, *adhE*, and *adhP* are deleted—from which the lactate dehydrogenase *ldhA* was deleted. The strain was adapted to the biological feedstock, as described in Section 2.3.1, prior to its use in flask and bioreactor experiments. The adapted strain stocks were stored in 25% *v*/*v* pure glycerol at −80 °C and plated on agar/biological feedstock plates prior to each broth culture. A single colony was inoculated in tryptic soy broth (TSB) and the 2nd pre-culture was performed either in M9 minimal media or in biological feedstock, or in a mixture of them. Typical M9 media composition was 6.8 g Na_2_HPO_4_, 3.0 g KH_2_PO_4_, 1.0 g (NH_4_)_2_SO_4_, 0.5 g NaCl, 4 mL of 0.1 M MgSO_4_ × 7 H_2_O, 100 μL of 1 M CaCl_2_ × 2 H_2_O, and 1 mL of a SL-10 trace element solution per liter of deionized water. The SL-10 solution was composed of 1 mL 25% *v*/*v* HCl, 1.5 g FeCl_2_ × 4 H_2_O, 70.0 mg ZnCl_2_, 100 mg MnCl_2_ × 4 H_2_O, 3.0 mg H_3_BO_3_, 190 mg CoCl_2_ × 6 H_2_O, 2.0 mg CuCl_2_ × 2 H_2_O, 24 mg NiCl_2_ × 6 H_2_O, and 36 mg Na_2_MoO_4_ × 2 H_2_O, per liter of deionized water. The medium was sterilized in all cases in an autoclave (Raypa Steam Sterilizer, Rayapa, Barcelona, Spain) at 121 °C for 20 min.

### 2.2. Biological Feedstocks

The LA fermentation broth was provided by the Chemical Engineering Department, Technical University of Denmark (DTU), for use as feedstock to the bio-purification process. A mixture of candy waste and digestate was used for LA production, as described by Papadopoulou, Vance et al. [28]. Preliminary centrifugation was performed to remove cells and suspended solids [28], and the initial feed was analyzed via HPLC; its composition is presented in Table 2.

The initial feed stream was treated through a MF step, as described by Papadopoulou, González et al. [5]; then, it was centrifuged 3 times at 10,000× *g* for 20 min and autoclaved at 121 °C for 20 min. Given that the concentration of maltose was very high compared to the typical concentration of simple sugars in UF permeates, as presented in Table 1, the initial feed was diluted at a ratio 1:10 with deionized water (to reach a typical concentration of maltose approx. 2–3 g/L) and the addition of LA to reach the targeted concentration of 10 g/L that did not inhibit the *E. coli* strain growth [20] and AA to simulate the characteristics of UF permeate streams, as listed in Table 1 (feedstock F1). Feedstock F1 was used for all the subsequent experiments concerning cell adaptations, validations in the flask, scale-up in bench-scale bioreactor, and MBR process development.

Concerning the 2nd feedstock (Feedstock F2), an NF step was implemented in a lab-scale cross-flow filtration pilot unit (Figure S1). A commercially available module of hollow fiber membranes was used (MP025 dNF80, NX Filtration, Netherlands) for the NF process. The total active surface of the inside-out module was 0.05 m^2^, cross-flow was set at 2 L/min, and the average transmembrane pressure, measured in both the inlet and outlet of the membrane module, was approx. 5.2 bar. The initial feed stream was centrifuged 3 times at 10,000× *g* for 20 min. The supernatant was autoclaved at 121 °C for 20 min and then filtered through the NF membrane module employing a diafiltration process mode. To meet the specifications of the NF membrane module concerning the suspended solids, a 1:5 feed/diafiltrate ratio was used, employing deionized water as the diafiltrate. In particular, the concentration of total suspended solids (TSSs) of the diluted feedstock was specified at 69.5 mg/L. The performance of the NF process was stable, and membrane permeability was between 1.5 and 2.0 L/m^2^·h·bar, reaching a final recovery ratio of approx. 70% (Figure S2). The NF permeate was practically transparent since the majority of the colored compounds were successfully rejected by the NF membrane (Figure S3).

LA was added to the NF permeate to reach the targeted concentration of 10 g/L that did not inhibit *E. coli* strain growth [20], and AA was added to simulate the composition of the NF permeate of J Ecker et al. [18] (Table 1). Feedstock F2 was used for MBR process development and was re-autoclaved at 121 °C for 20 min prior to each use. The compositions of the initial feed stream from DTU and of the two feedstocks in this study are presented in Table 2.

### 2.3. Experimental Design

#### 2.3.1. Adaptation of the *E. coli* A1:ldhA Strain on the Biological Feedstock

**Figure 2:**
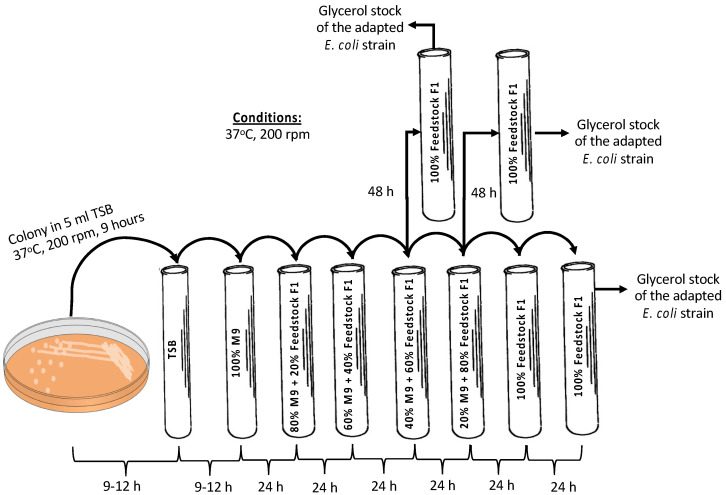
Adaptation methodology for the growth of *E. coli* A1:ldhA strain on feedstock F1.

As already shown [20], *E. coli* A1:ldhA cells are capable of selectively catabolizing carbon sources, i.e., glucose and AA, without catabolizing LA. Feedstock F1 was used to confirm that the *E. coli* A1:ldhA strain is able to grow on such feedstock; an adaptation method followed, starting from 100% synthetic M9 media supplemented with 0.5 g/L glucose, 0.5 g/L AA, and 5 g/L LA as the substrate to a final substrate comprising 100% feedstock F1. The methodology is schematically presented in Figure 2. A single colony of the *E. coli* strain was picked from tryptic soy agar (TSA) plate and initially inoculated in 5 mL tryptic soy broth (TSB) culture media. In total, 4% *v*/*v* of the grown cells in TSB were inoculated in 100% synthetic media (i.e., M9 minimal media supplemented with 5 g/L glucose, 0.5 g/L AA and 5 g/L LA) as the substrate in 16 mL glass tubes. After 24 h of fermentation, 4% *v*/*v* of the culture was inoculated in 5 mL fresh culture media composed of 80% synthetic media and 20% real feedstock F1. Sequential inoculation of 4% *v*/*v* cultivation was repeated every 24 h or 48 h by increasing 20% the part of biological feedstock and, respectively, decreasing the synthetic media aiming at the growth of the strain solely (i.e., 100%) on the feedstock F1. Re-inoculation in 100% feedstock F1 was tested along with direct inoculation from 40:60 M9/feedstock F1 to 100% feedstock F1, and from 20:80 M9/feedstock F1 to 100% feedstock F1. The cells grown on 100% feedstock F1 of each tested case were stored in 25% *v*/*v* pure glycerol at −80 °C and plated on agar/biological feedstock plates for further use.

#### 2.3.2. Preliminary Validation in Shake Flask Scale

Preliminary tests were performed to optimize the growth protocol on feedstock F1 by employing two different pre-culture steps. In the 1st case, a single colony from the adapted cells was initially inoculated in 5 mL TSB. Then, 250 mL shake flasks, containing 100 mL of feedstock F1, with 4 mL (4% *v*/*v*) inoculum, were incubated at 37 °C and 200 rpm. In the 2nd case, the single colony of the adapted cells was directly inoculated in 5 mL of feedstock F1 before inoculating the 250 mL shake flask. These tests aimed to assess the effect of the different pre-culture steps on cells’ growth in flasks as well as to confirm the ability of the cells to grow on feedstock F1 by selectively consuming the impurities (i.e., maltose and AA). The shake flask experiments were performed in biological triplicates.

#### 2.3.3. Scale-Up in a Bench-Scale Bioreactor

**Figure 3:**
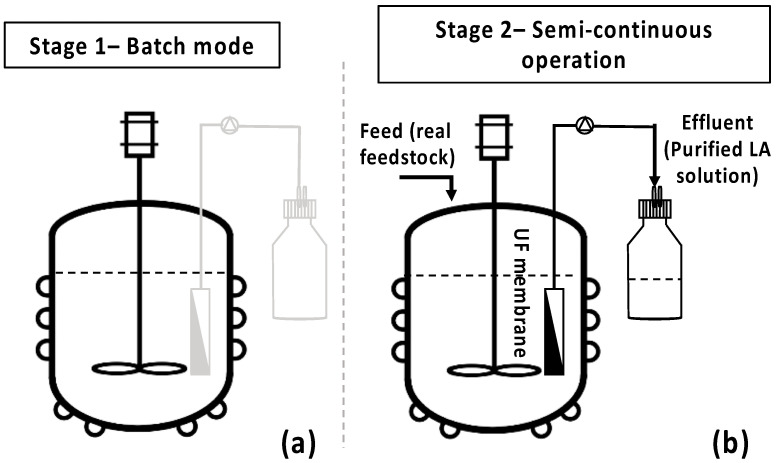
Batch and semi-continuous membrane bioreactor (MBR) experimental set-up. (**a**) Bench-scale bioreactor under batch operation; (**b**) semi-continuous operation of the MBR.

The bio-purification process was scaled-up in a 3 L bench-top bioreactor (BioFlo^®^ 120, Eppendorf, Hamburg, Germany), aiming to validate the selective removal of impurities in the presence of LA in larger-scale experiments (Figure 3a). Two similar fermentation batch tests of 1.8 L feedstock F1 final volume were performed under pH, temperature, agitation, and airflow control, while growth was monitored by an optical density probe (Hamilton Dencytee Unit 225, Hamilton Co., Reno, NV, USA), and dissolved oxygen (DO) was recorded by an optical DO sensor (Eppendorf ISM^®^ probe Eppendorf, Hamburg, Germany). pH was regulated at 7.0 by an automatic addition of 2.5 N NaOH and 2.5 N H_2_SO_4_ solution. Agitation speed was set at 450 rpm, temperature at 37 °C, and airflow at 1.5 vvm. A single colony from the adapted cells was initially inoculated in 5 mL TSB. Then, 250 mL shake flasks, containing 100 mL of feedstock F1, were inoculated with 4 mL (4% *v*/*v*) inoculum overnight. The bioreactor was inoculated with 4% *v*/*v* of the 2nd pre-culture after 22 h cultivation. Fermentation lasted for 48 h, aiming to complete maltose and AA depletion. AA and maltose consumption rates were evaluated for the subsequent design and development of the semi-continuous MBR bio-purification process. The batch experiments were repeated twice with hourly sampling and a 12 h difference swift in the start of the sampling in order obtain data during the whole 48 h fermentation period.

#### 2.3.4. Semi-Continuous MBR Bio-Purification Process for LA—Comparative Assessment

Τo assess the efficiency of the integrated MBR bio-purification process, the developed methodology as described by Nastouli et al. [20] was followed, comprising two stages. Stage 1 comprises the batch operation of the bioreactor (Figure 3a), while stage 2 (Figure 3b) is an intermittent one with a custom-made UF membrane module [29] used to draw the treated effluent while retaining the active cells in the bioreactor, leading to a semi-continuous process operation mode [20].

Feedstocks F1 and F2 were used for the MBR experiments. Feedstock F1 was used for three MBR experiments, i.e., MBR 1, MBR 2, and MBR 3. Feedstock F2 was used for experiment MBR 4 to comparatively assess the efficiency of the MBR bio-purification process. The protocol described in Section 2.3.3 for the batch fermentation experiments was applied in the 1st stage of the MBR bio-purification process. The goal of the 1st stage was to achieve high cell biomass as well as total depletion of the impurities contained in the initial volume of the biological feedstock while retaining the LA concentration constant. Afterwards, the 2nd stage of the process was conducted, which comprised several permeate/feed cycles, aiming to achieve total depletion of impurities (i.e., AA and maltose) before the next cycle of the permeate/feed. The operating conditions of each MBR experiment are presented in Table 3.

### 2.4. Analytical Methods

#### 2.4.1. High-Performance Liquid Chromatography (HPLC)

A method was set up for the determination of sugar concentration in an HPLC Shimadzu LC-10AT VP Liquid Chromatograph (Shimadzu Co., Kyoto, Japan) using a ShodexTM Sugar SH1011 (8.0 mm I.D. × 300 mm) column and a Shimadzu Refractive Index Detector RID-10A. Organic acids were measured by the same method and column using a Shimadzu SPD-M20A UV detector. Each fermentation broth sample was centrifuged at 4500 rpm for 7 min and the supernatant was diluted accordingly, and it was filtered through 0.45 μm filters. The mobile phase was 5 mM H_2_SO_4_, the flow rate was set to 0.4 mL/min, and the oven temperature at 60 °C. Concerning statistical analysis, average values of experimental measurements of the samples (triplicates) and their standard deviations (SDs) were calculated through Microsoft Excel 2013 software (Microsoft Corporation, Washington, DC, USA).

#### 2.4.2. Total Organic Carbon (TOC) and Total Nitrogen (TN)

TOC and TN were measured with a Shimadzu TOC-5000A TOC/TN Analyzer. Calibration curves of 10–100 ppm and 0.5–5 ppm were used for the TOC and the TN measurements, respectively. Each fermentation broth sample was centrifuged at 4500 rpm for 7 min, and the supernatant was diluted accordingly. The analyses were performed in triplicates. The presented results have a SD of less than 5%.

#### 2.4.3. Optical Density (OD_600_) and Dry Biomass

To define the cell growth, optical density was measured at 600 nm by a photometer (UV1700 Pharmaspec Shimadzu UV-VIS Spectrophotometer, Shimadzu Co., Kyoto, Japan) during fermentation experiments in flasks and in MBR experiments. For batch bioreactor experiments, OD_600_ was also measured on-line through a Dencytee Arc (Hamilton Co., Reno, NV, USA) cell density sensor. Cell density sensor data were recorded every 6 or 12 min. A correlation of OD_600_ with dry biomass was developed by regular analysis of the dry biomass concentration; several fermentation samples of 10 mL volume were collected and centrifuged at 4500 rpm for 7 min. The supernatant was discarded, the remaining cell pellet was resuspended in 1 mL H_2_O, and centrifuged again under the same conditions. The cells were double-washed and dried at 60 °C until they reached constant weight.

## 3. Results and Discussion

### 3.1. Adaptation of the E. coli A1:ldhA Strain on the Biological Feedstock

*E. coli* strain adaptation in feedstock F1 was assessed by measuring the growth of the cells through OD_600_ measurements. It was considered that when OD_600_ exceeds 1.00, the *E. coli* strain grows satisfactorily. According to Table 4, up to the ratio 40% M9 minimal media to 60% feedstock F1 in the culture media, OD_600_ exceeded 1.00 within 24 h. For cultures composed of 20% M9 minimal media and 80% feedstock F1, the growth was delayed, and OD_600_ exceeded 1.00 at 48 h. The same trend was observed for the culture media composed of 100% feedstock F1 that was inoculated by a strain adapted to 40:60 M9/feedstock F1 or to 20:80 M9/feedstock F1. Re-inoculation to 100% feedstock F1 culture media from 100% feedstock F1 inoculum achieved OD_600_ values 1.210 ± 0.015 within 24 h. In addition, maltose, glucose, AA, and LA concentrations were recorded. LA remained constant in all adaptation steps. Maltose and glucose were the main sugars present in the culture media and they were both sufficiently consumed during the adaptation strategy. It is known that the *E. coli* strain follows a diauxic growth pattern and that the consumption of sugars is prioritized. Therefore, AA was used by the cells as a secondary available carbon source. Table 4 shows that AA was not sufficiently consumed; in fact, it was excreted in most cases, especially when glucose was still present. For instance, for 100% feedstock F1 culture, glucose was totally consumed within 24 h while 0.82 ± 0.05 g/L maltose was still available. At this time point, the AA concentration increased from 3.65 ± 0.014 g/L at t = 0 h to 3.75 ± 0.04 g/L at t = 24 h, while 0.75 g/L AA was roughly consumed from 24 to 48 h. Concurrently, 0.20 g/L of maltose was consumed as well. The aforementioned results show that the *E. coli* strain is capable of removing glucose, maltose, and AA, but not LA. However, when the fermentation test stopped (i.e., when a culture with an OD_600_ > 1.00 is obtained), AA uptake is not finished, given *E. coli*’s metabolic preference.

### 3.2. Preliminary Validation in Shake Flask Scale

**Figure 4:**
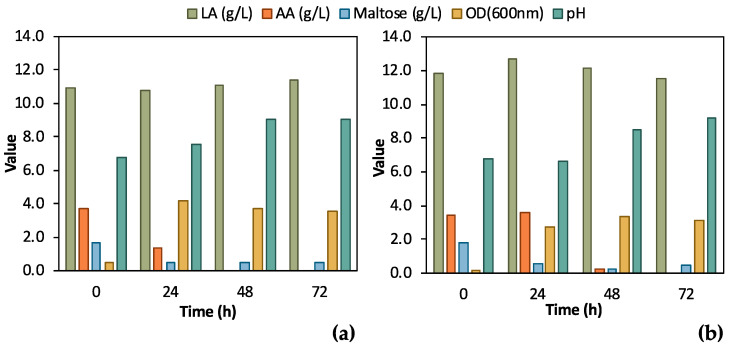
Comparison of results from flask fermentation tests to assess the effect of 1st pre-culture step; (**a**) single colony was inoculated in tryptic soy broth (TSB); (**b**) single colony was inoculated in 100% feedstock F1.

A single colony of the adapted *E. coli* strain was inoculated in 5 mL TSB media or in 5 mL feedstock F1 to assess the effect of the differently grown inoculum on bio-purification in shake flask tests. The results from these preliminary tests, which are illustrated in Figure 4, clearly validate the bio-purification of feedstock F1 in shake flask tests. Indeed, LA remained constant in both cases, while AA and maltose were practically consumed. AA was totally consumed both when TSB and biological feedstock were used for the inoculum, whereas maltose was partly consumed. In the case of feedstock F1 inoculum, AA consumption was delayed for 24 h compared to the inoculum in TSB. In addition, a stronger culture was obtained when TSB was used as the medium for inoculum growth; the OD_600_ value reached 4.15 ± 0.13 at t = 24 h compared to OD_600_ 3.35 ± 0.10, which was recorded as the highest OD_600_ value in the case of feedstock F1 inoculum, at t = 48 h. pH increased as expected, due to the consumption of AA, exceeding 9.0 for both shake flask tests.

According to Figure 4, during the initial bio-purification shake flask experiments, successful and reasonable removal of organic carbon impurities such as maltose and AA was demonstrated. The preliminary comparative assessment demonstrated that TSB inoculum slightly outmatched the feedstock F1 inoculum; therefore, TSB was used for the subsequent experimental steps. Easier handling of TSB due to consistent composition and properties as culture media is another advantage compared to real feedstock, which may differ depending on its origin. The fluctuating composition of the feedstock F1 can significantly affect inoculum growth and, as a result, the subsequent steps of the process. The conditions of the pre-culture step should be constant to assess the efficiency of the bio-purification process itself.

### 3.3. Scale-Up in a Bench-Scale Bioreactor

**Figure 5:**
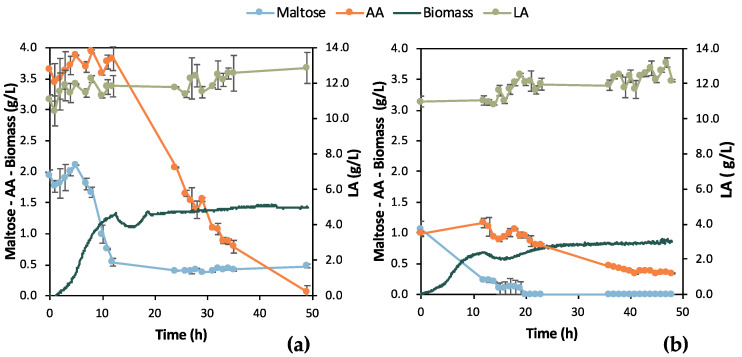
Results from two batch fermentation/bio-purification processes with feedstock F1 in a bench-scale bioreactor with 12 h swift sampling: maltose, AA, biomass, and LA concentration temporal profile; (**a**) Batch 1; (**b**) Batch 2.

Batch fermentation experiments in a bench-top bioreactor were designed according to the results obtained from the shake flask tests. Two similar batches were run for 48 h aiming at total depletion of maltose and AA. Samples were collected with a 12 h difference swift from the 1st batch experiment to the 2nd to collect kinetic data for bio-purification in the bench bioreactor. However, the initial composition of the two F1 feedstock batches was different, which likely led to the difference between the results of the two experiments presented in Figure 5. In particular, the initial maltose concentration was almost double in Batch 1 (i.e., 1.94 ± 0.09 g/L) compared to Batch 2 (i.e., 1.04 ± 0.13 g/L) and corresponded to almost a double OD_600_ value, i.e., double cell growth. However, both growth curves followed a similar trend, i.e., the lag phase lasted for 5 h and then cells grew exponentially up to t = 13 h of fermentation, roughly. Another difference of great importance is that AA was totally depleted in the 1st batch, which was not achieved in the 2nd batch. The opposite happened concerning maltose. Maltose consumption was apparently hindered in the 1st batch, whereas it was fully consumed in the 2nd batch. LA did not seem to be consumed despite some variations in the 1st batch (SD = 0.82 g/L) and in the 2nd batch (SD = 0.63 g/L), with 11.70 g/L and 11.98 g/L LA mean concentrations, respectively.

**Figure 6:**
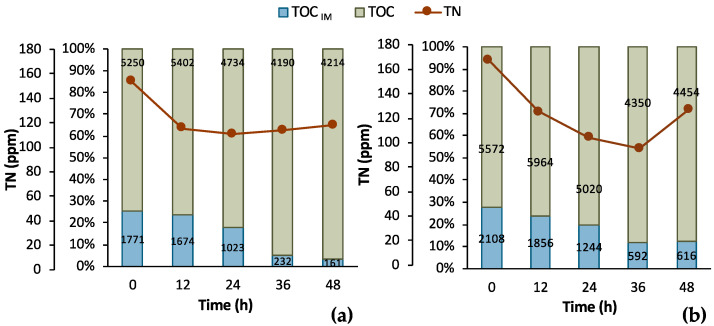
Results from 2 batch fermentation/bio-purification processes with feedstock F1 in a bench-scale bioreactor with 12 h swift sampling; (**a**) Batch 1; (**b**) Batch 2; total organic carbon (TOC), total organic carbon of impurities (TOM_IM_); total nitrogen (TN).

To further assess the process, supplementary data are illustrated in Figure 6 regarding the TOC and TN measurements. It is obvious that a sufficient amount of nitNrogen, higher than 100 ppm, was present in both cases. A significant amount of TOC was removed in both cases, as well. In particular, the total TOC concentration minus the concentration of LA corresponds to the TOC concentration of the impurities (TOC_IM_) in the biological feedstock. TOC_IM_ reduced from 1771 ppm to 161 ppm within 48 h in Batch 1 and from 2108 ppm to 616 ppm in Batch 2 in same time period. The recorded data are very encouraging for a further investigation and development of the LA bio-purification process in an MBR system.

### 3.4. Semi-Continuous MBR Bio-Purification Process for LA—Comparative Assessment

The next step of the current study was to evaluate the performance of an integrated MBR process based on the collected data from the batch fermentation experiments in the bioreactor. The process was divided in two main parts, the initial batch fermentation process (1st stage), followed by the operation of the bioreactor combined with the UF membrane (MBR concept), when the bench-scale bioreactor was operated in a semi-continuous mode (2nd stage), with cycles of withdrawing treated substrate and the addition of fresh feedstock. MBR 1, MBR 2, and MBR 3 experiments were conducted with feedstock F1, which simulated a UF permeate. Due to the use of real feedstock, some differences were recorded between the different batches; however, their composition was practically similar concerning LA, AA, and maltose.

**Figure 7:**
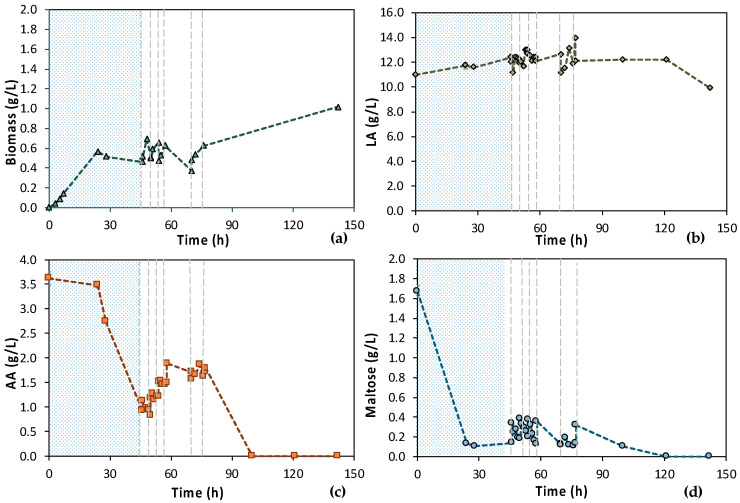
Concentration profiles of (**a**) biomass; (**b**) LA; (**c**) AA; and (**d**) maltose of the initial (MBR 1) semi-continuous MBR bio-purification performed with feedstock F1. The textured part corresponds to the 1st stage of the process; dash lines correspond to the beginning of each cycle of the 2nd stage of the process.

According to Figure 7, the 1st stage of MBR 1 lasted for 46 h, aiming at total depletion of AA and maltose. Figure 7a also shows that cells were growing exponentially until 24 h, concurrently with the maltose uptake (Figure 7d). Then, there was a stationary phase and a slight decay, even though a significant amount of AA was consumed, approx. 2.50 g/L (Figure 7c). However, it seems that more time was needed by the cells to totally consume the AA, since AA removal during the 1st stage reached approx. 75%. Concerning maltose, 92.2% maltose removal was achieved within 24 h and remained constant until the end of the batch operation (t = 46 h); 0.11 ± 0.01 g/L maltose was recorded at t = 24 h and 0.14 g/L ± 0.00 at t = 46 h (Figure 7d).

**Figure 8:**
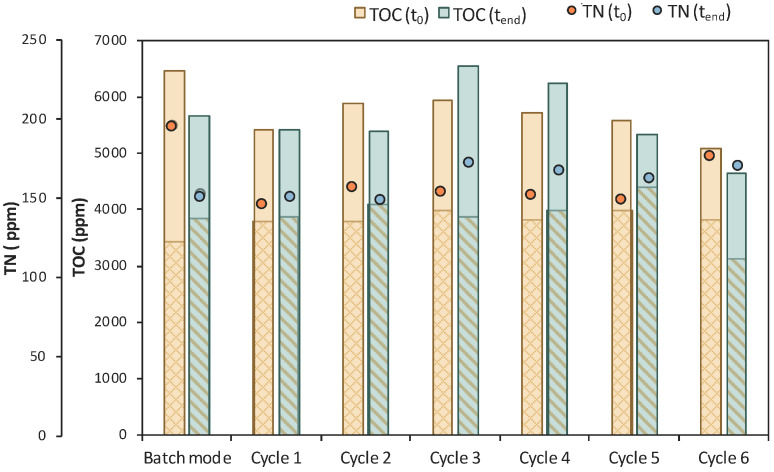
TOC measurements of MBR 1 in the beginning (yellow bar) and in the end (green bar) of batch stage 1 and at each cycle of stage 2 of the process; the textured part of the bars corresponds to the TOC concentration of LA in ppm; TN measurements in the beginning (orange dot) and in the end (blue dot) of batch stage 1 and at each cycle of stage 2 of the process.

Six (6) cycles of membrane filtration/fresh feedstock were conducted during the 2nd stage of the process. It can be stated that the time intervals between each UF step were quite short, mainly for the AA consumption, which was not actually consumed. Instead, AA increased after each new feed. Sugars were preferentially used by the *E. coli* cells as expected; still, maltose was not totally consumed. In general, it seems that an inhibitory factor negatively affected the whole process. In addition, after 120 h of fermentation, LA consumption was observed (Figure 7b), which probably means that the culture was contaminated. TOC and TN were also measured to gain a better insight into the process’s efficiencies. Figure 8 depicts the measurements of TOC and TN at the beginning and at the end of the 1st stage as well as of each cycle of 2nd MBR bio-purification stage. A significant part of the total TOC corresponds to the LA contained in the feedstock, which remained constant until the end of cycle 6. During batch operation (till t = 46 h), the TOC concentration of the other organic substances apart from LA decreased by approx. 1211 ppm, which was removed during the 1st stage. However, during cycle 3 and 4 of the 2nd stage, TOC was increased by the end of each cycle, possibly denoting cell lysis and the release of intracellular material in the fermentation broth. TN measurements follow a similar trend, further supporting this hypothesis. During the last cycle (cycle 6) of MBR bio-purification, TOC decreased together with the LA concentration, which supports the hypothesis of a potential contamination of the fermentation culture.

**Figure 9:**
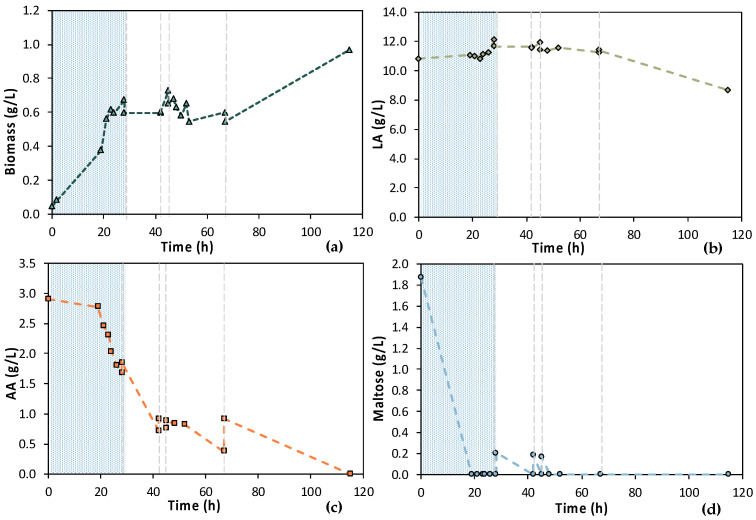
Concentration profiles of (**a**) biomass; (**b**) LA; (**c**) AA; and (**d**) maltose of the second (MBR 2) semi-continuous MBR bio-purification performed with feedstock F1; the textured part corresponds to the 1st stage of the process; dash lines correspond to the beginning of each cycle of the 2nd stage of the process.

Although the TN concentration seems to be sufficient, a potential inhibitory factor could be the lack of other macronutrients, as it has been observed in a previous study [20]. Therefore, the macronutrient concentration was doubled in the MBR 2 experiment compared to MBR 1, and a different experimental design was followed for both stages of MBR bio-purification. These changes include the 1st stage lasting 24 h in MBR 2 (Figure 9) compared to 46 h in MBR 1, whereas longer time intervals were applied for the 2nd stage of the process. Total maltose removal (Figure 9d) and 42.2% AA removal (Figure 9c) were achieved during the 1st stage. Four cycles of adding the feedstock F1 followed, as depicted in Figure 9.

During semi-continuous operation, the fermentation broth was free of maltose at the end of each cycle, whereas AA removal was not complete. In particular, from 45 to 67 h (i.e., within 22 h), only ~0.50 g/L of AA was removed, while the concentration of the maltose was practically zero. Once again, LA consumption was observed at a longer fermentation time, i.e., from 67 to 115 h of fermentation (Figure 9b). In this time period, i.e., the final cycle of the MBR bio-purification (cycle 4), AA total removal, and doubling of biomass concentration were observed, which are in accordance with the hypothesis of a potential contamination.

**Figure 10:**
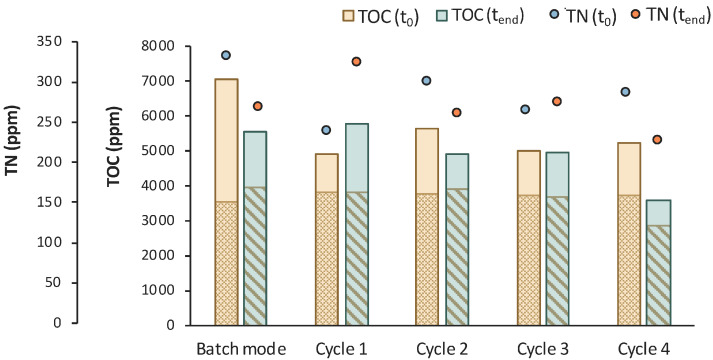
TOC measurements of MBR 2 in the beginning (yellow bar) and in the end (green bar) of batch stage 1 and at each cycle of stage 2 of the process; the textured part of the bars corresponds to the TOC concentration of LA in ppm; TN measurements in the beginning (orange dot) and in the end (blue dot) of batch stage 1 and at each cycle of stage 2 of the process.

Concerning TN, it can be verified that there was no nitrogen limitation, as it remained >200 ppm during the whole process (Figure 10). A significant drop of the TOC concentration was also observed during the 4th cycle of the 2nd stage, which is in accordance with the LA consumption. Concerning AA and maltose removal, process efficiency was generally improved in MBR 2, but the desired goals were not achieved. A low initial concentration of the available carbon sources might be a potential limiting factor of the MBR process, and LA consumption, when longer fermentation times are applied, implies that a potential contamination occurs.

**Figure 11:**
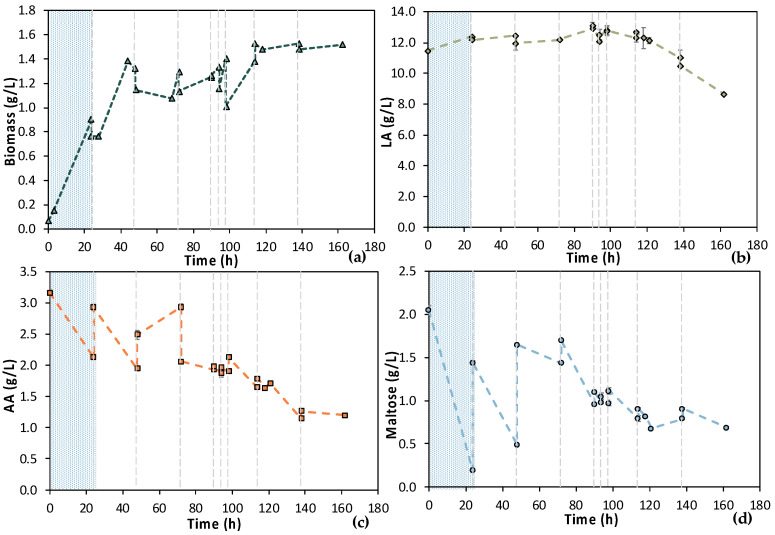
Concentration profiles of (**a**) biomass; (**b**) LA; (**c**) AA; and (**d**) maltose of the semi-continuous MBR bio-purification performed with feedstock 1 (MBR 3); the textured part corresponds to the 1st stage of the process; dash lines correspond to the beginning of each cycle of the 2nd stage of the process.

To further assess the MBR bio-purification process, especially during the latter fermentation stages, the MBR 3 experiment, which was quite similar to the previous MBR 2 experiment, was performed. The 1st stage of MBR 3 lasted for 24 h and was followed by the 2nd stage, which included six cycles of adding feedstock F1, as shown in Figure 11; the total duration of the experiment was 160 h. During this experiment, a similar behavior to the previous experiment was observed, i.e., the consumption of maltose and AA was not complete at any stage of MBR bio-purification. The LA concentration was significantly reduced from 12.67 ± 0.11 g/L to 8.66 ± 0.12 g/L during cycle 7 and 8 (from t = 114 h to t = 162 h), which depicts clear evidence of a potential contamination, considering that the *E. coli* strain used in this study is incapable of consuming LA.

**Figure 12:**
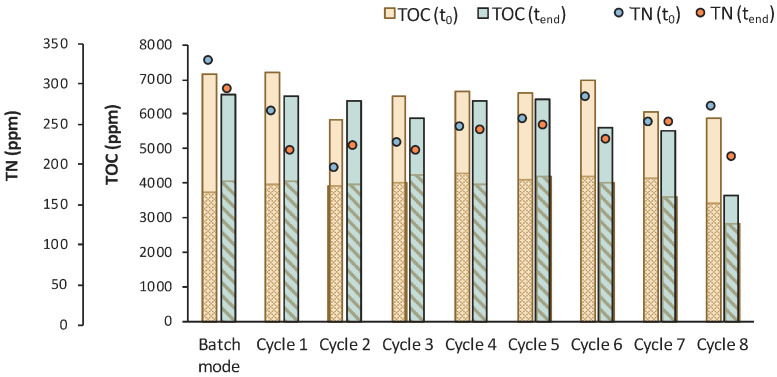
TOC measurements of MBR 3 in the beginning (yellow bar) and in the end (green bar) of batch stage 1 and at each cycle of stage 2 of the process; the textured part of the bars corresponds to the TOC concentration of LA in ppm; TN measurements in the beginning (orange dot) and in the end (blue dot) of batch stage 1 and at each cycle of stage 2 of the process.

The assumption of a potential contamination is also verified by the TOC and TN measurements presented in Figure 12. The reduction in the TOC_IM_ that is the concentration of the impurities was insignificant, apart from cycles 1 and 3, where it was reduced by 746 and 843 ppm, respectively. A significant drop in TOC was observed during cycle 8, together with a reduction in the LA concentration. LA was continuously consumed after t = 120 h of fermentation and correlated with a drop in TN during the last cycle. These observations lead to the conclusion that the culture seemed to be contaminated at longer fermentation times.

The repeated contamination at longer fermentation times of every MBR experiment with biological feedstock F1 leads to the conclusion that the culture is not contaminated by accident. The initial feed stream is the LA fermentation broth comprising a mixed waste stream of 50% candy waste and 50% digestate. Digestate is a waste with many different microorganisms within [30,31], containing bacteria species which are resistant to high temperatures. In particular, spore-forming microorganisms may be resistant during (insufficient) heat sterilization, and these spore-forming microorganisms, when found at favorable conditions, can grow excreting toxic substances [32]. Different microorganisms of those present in the digestate exhibit these characteristics, such as some species of *Bacillus* genus [32,33]. The presence of these spores and their toxins produced have been reported to affect the *E. coli* cells’ growth and activity [34]. Moreover, at longer fermentation times, the spores may start to grow and contaminate the MBR process. Therefore, a critical issue for the development of the MBR bio-purification process is to use a different feedstock that could be free of any potential microbes or spores. As already mentioned, the initial feed stream was microfiltered in non-sterile conditions; thus, the existence of microbes or spores in the equipment and as a result in the feedstock is possible. Membrane filtration with a tight UF or NF membrane is capable of removing not only the spores but also any excreted toxins that are potentially produced during sporulation [34]. For this reason, feedstock F2 (after NF separation) was used for the last MBR experiment (MBR 4). Through the NF, discoloration of the biological feedstock was achieved (NF permeate), which indicates the removal of colored organic substances. Regarding its physicochemical characteristics, electrical conductivity (eC) was 1675 μS/cm, and the value of pH was 6.0.

**Figure 13:**
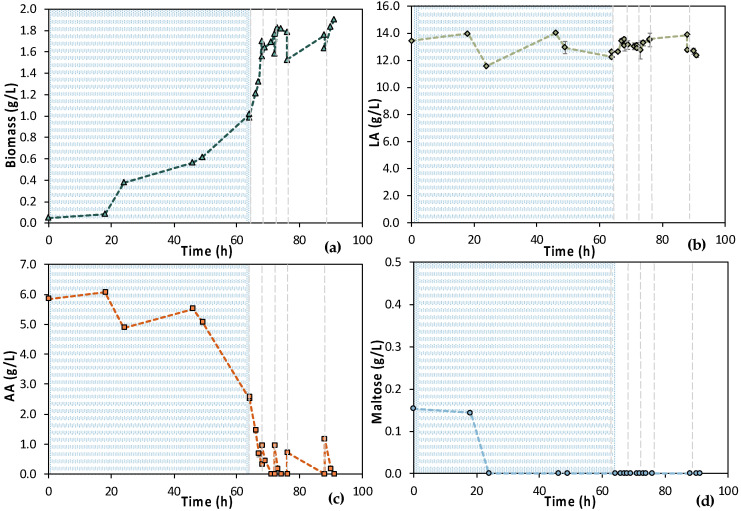
Concentration profiles of (**a**) biomass; (**b**) LA; (**c**) AA; and (**d**) maltose of the semi-continuous MBR bio-purification performed with feedstock F2 (MBR 4). The textured part corresponds to the 1st stage of the process. Dash lines correspond to the beginning of each cycle of the 2nd stage of the process.

Due to the NF membrane’s pore size, most of maltose (MW = 342.3 g/mol) contained in the initial feed stream was retained. On the contrary, the molecular size of AA (MW = 60.05 g/mol) and LA (MW = 90.08 g/mol) allowed them to pass through the membrane. Therefore, the composition of feedstock F2 differed compared to feedstock F1; the measured maltose concentration was below 0.5 g/L, while AA around 6.0 g/L. Results from MBR 4 experiment are presented in Figure 13. Concerning the 1st stage of the process, cells were not growing until 24 h of fermentation; macronutrients were added in the vessel to confront this potential limiting factor. However, cells’ growth was still limited; thus, trace elements contained in M9 minimal media were also added at t = 46 h. The potentially lack of trace elements, which could be partially retained by the NF membrane, seemed to be the limiting factor since cells started growing, after the addition of trace elements, reaching OD_600_ = 2.0, while AA was decreased from 5.51 ± 0.01 g/L at t = 46 h to 2.52 ± 0.02 g/L at t = 64 h. The 2nd stage of the process started at t = 64 h of fermentation and operated in a semi-continuous mode for 27 h. During the 1st cycle, the AA concentration dropped from 2.58 ± 0.01 g/L down to 0.31 ± 0.00 g/L within 4 h, while biomass increased from 0.98 ± 0.04 g/L to 1.70 ± 0.09 g/L. For the subsequent cycles, AA was totally depleted within the applied time intervals of fresh feedstock feeding. The low concentration of maltose that was within in the feedstock F2 resulted in its total consumption within 24 h of fermentation, during stage 1. During stage 2, maltose content was very low and was subsequently consumed quickly, but it was not detected by HPLC analysis. Therefore, a cell-free LA solution was obtained as the permeate of the UF membrane module of the MBR that did not contain any maltose or AA. It seems that trace elements and macronutrients contained in the initial feed stream were removed during the NF step, resulting in their insufficiency regarding cell growth. It can also be concluded that undesirable microorganisms, spores (and potential toxins), were removed through the NF step since no contamination took place, even after 90 h of fermentation.

**Figure 14:**
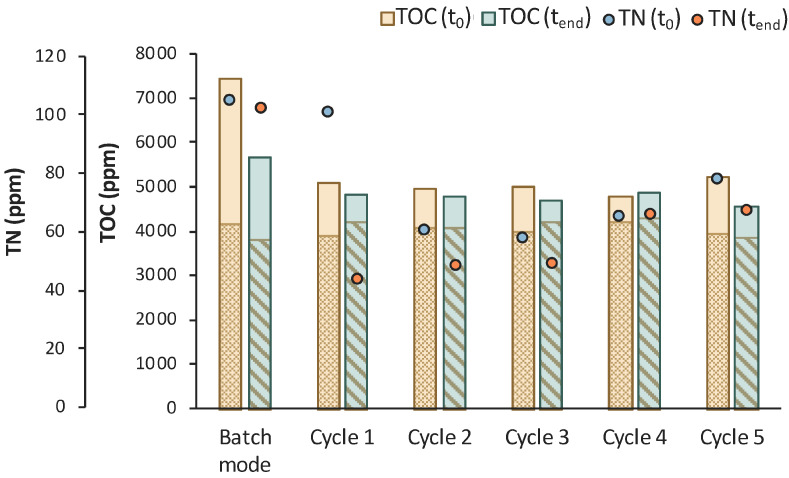
TOC measurements of MBR 4 in the beginning (yellow bar) and in the end (green bar) of batch stage 1 and at each cycle of stage 2 of the process; the textured part of the bars corresponds to the TOC concentration of LA in ppm; TN measurements in the beginning (orange dot) and in the end (blue dot) of batch stage 1 and at each cycle of stage 2 of the process.

According to Figure 14, total TOC concentration decreased from 7450 ppm to 5684 ppm during the 1st stage of MBR bio-purification. A reduction was also observed for every cycle of the permeate/fresh feed of feedstock F2. It is important to state that when excluding the concentration of the TOC that corresponds to LA, the impurities were significantly reduced during the whole process.

Furthermore, critical metrics of the MBR experiments were calculated to assess the efficiency of the semi-continuous MBR bio-purification process (Table 5). Maximum AA consumption was increased from 0.058 g/L/h in MBR 1 to 0.587 g/L/h in MBR 4. The maximum consumption rate and the maximum percentage of removal of both AA and maltose correspond to the highest value of the consumption rate and were calculated separately for each filtration/feed cycle of the respective MBR test. The same methodology was followed for the calculation of the maximum (absolute) value of transmembrane pressure (TMP), which was below 0.1 bar for MBR 1 and MBR 4 (72 mbar and 45 mbar, respectively), whereas it reached almost 0.5 bar for MBR 3 and approx. 1 bar for MBR 4. The net recovery rate reached 44.29 mL/h for MBR 4, i.e., it was almost 4 times higher than the net recovery rate of MBR 1, MBR 2, and MBR 3 experiments. Finally, the maximum removal ratio of both maltose and AA reached 100% for MBR 4.

### 3.5. UF Membrane Performance

**Figure 15:**
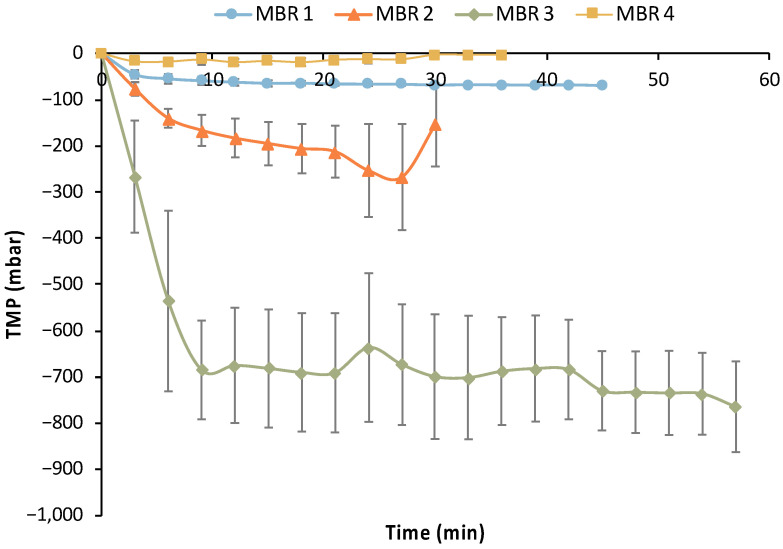
Average trans-membrane profile (TMP) profile of the filtration cycles of the four MBR experiments.

In addition to the efficiency of removing maltose and AA (main impurities) by *E. coli* cells, the performance of the UF membrane module is crucial for the MBR bio-purification process. Figure 15 summarizes the transmembrane pressure (TMP) temporal variation for each filtration cycle of all MBR experiments, which is indicative of the UF membrane fouling.

Limited fouling was observed during the 1st MBR experiment based on the data presented in Table 5 and Figure 15, i.e., the maximum TMP drop reached −72 mbar. However, a sharp reduction in average TMP values was observed in MBR 2 and especially in the MBR 3 experiment, reaching −0.7 bar. Thus, it is clear that significant fouling occurred in the MBR 2 and (especially) in MBR 3 experiments. The same custom-made membrane module was used in all these MBR experiments after the implementation of a standard chemical cleaning protocol; the latter comprised a rinsing with clean DI water for at least 1 h, followed by cleaning with 200 ppm NaOCl solution for 1.0 h, a second rinsing with DI water to remove any traces of the NaOCl, and then a typical sterilization step. The reusability of the UF membrane was positively assessed concerning its performance without any significant negative effect in a previous study [29]. However, the characteristics of the biological feedstock used in the present study and the potential growth of other bacteria species (e.g., of *Bacillus* genus), which have the ability to produce biofilms [35], have probably led to the deterioration of the membrane’s filtration performance. This assumption is also supported by the fact that the membrane’s filtration performance was totally restored (without any change in the preceding chemical cleaning protocol and the sterilization step) during the MBR 4 experiment (Figure 15), when practically no membrane fouling was observed. The insignificant fouling in MBR 4 is also reflected in the respective metrics of Table 5, i.e., the maximum TMP drop was only −45 mbar, which also supports the aforementioned assumption.

## 4. Conclusions

The engineered *E. coli* A1:ldhA strain was successfully adapted to 100% real/biological feedstock, originating from the fermentation of a mixture of candy waste and digestate from a shake flask to a bench-scale bioreactor level. Selective catabolism of maltose and AA was verified in larger-scale and more realistic operating conditions. The semi-continuous MBR bio-purification process was designed to verify the proof-of-concept using two different feedstocks. The feedstock that simulated the UF permeate was assessed as unsuitable for the bio-purification process since total depletion of AA and maltose was not possible. Concurrently, repeated contamination was observed, resulting in the consumption of LA, and significant membrane fouling was also observed. An NF process step was applied for removing any potential inhibitory/contamination factors contained in the substrate due to its origin (i.e., real digestate). In this case, macro- and micro-nutrients were supplemented in the bioreactor to achieve 100% removal of maltose and AA by the cells after consecutive feeding steps of biological feedstock. Fouling was very low, and an effluent free of cells, AA, and maltose was obtained.

The overall outcome of this study is that the MBR bio-purification process can be successful applied to real feedstocks from the fermentation of low-cost renewable agro-industrial feedstocks. However, specific pre-treatment steps should be applied, mainly for the removal of active cells, potential biological contaminants, and the bulk of other organic macromolecules. The bio-purification process seems to be better suited for less complex process streams, towards the final steps of a LA downstream purification strategy, mainly focusing on removing impurities with a high chemical affinity to LA that cannot be totally removed through conventional physicochemical processes and that are present in relatively low concentrations.

## Figures and Tables

**Table 1:** A summary of the composition of different membrane permeate streams in LA downstream processing from renewable, low-cost feedstocks.

Feedstock Description	Initial Feedstock	LA (g/L)	Acetic Acid (AA) (g/L)	Sugars (g/L)	Reference
UF permeate	Grass silage leachate	20.40	3.31	11.00	[17]
UF permeate	Grass silage leachate	28.00	4.98	8.90	[19]
UF permeate	Bio-pulp	15.40	1.01	n.d.	[6]
NF permeate	Grass silage leachate	33.80	7.81	0.17	[18]
NF permeate	Bio-pulp	14.70	1.02	- ^1^	[6]

^1^ not available.

**Table 2:** Composition of the initial feed stream and the feedstocks F1 and F2 used in this study.

Compound	Initial Feed (g/L)	Feedstock F1 (g/L)	Feedstock F2 (g/L)
LA (g/L)	29.04	~10	~10
Maltose (g/L)	35.60	~2.0	~0.5
AA (g/L)	0.55	~3.0	~6.0

**Table 3:** Experimental design of semi-continuous MBR experiments.

Description				MBR 1	MBR 2	MBR 3	MBR 4
General conditions			Feedstock type	Feedstock F1	Feedstock F1	Feedstock F1	Feedstock F2
			Nutrients in stage 1 (batch)	25% of M9 solution	50% of M9 solution	50% of M9 solution	100% of M9 solution + trace elements
			Nutrients in stage 2 (semi-continuous)	25% of M9 solution	50% of M9 solution	50% of M9 solution	100% of M9 solution + trace elements
Stage 1 (Batch mode)			Fermentation time	t = 46 h	t = 28 h	t = 24 h	t = 64 h
Stage 2 (Semi-continuous mode)	Cycle 1	1.1	Effluent/Feed volume	200 mL	200 mL	300 mL	230 mL
		1.2	Fermentation time	t = 4 h	t = 14 h	t = 24 h	t = 4 h
	Cycle 2	2.1	Effluent/Feed volume	200 mL	200 mL	300 mL	230 mL
		2.2	Fermentation time	t = 4 h	t = 3 h	t = 24 h	t = 4 h
	Cycle 3	3.1	Effluent/Feed volume	200 mL	200 mL	225 mL	230 mL
		3.2	Fermentation time	t = 4 h	t = 22 h	t = 18 h	t = 4 h
	Cycle 4	4.1	Effluent/Feed volume	300 mL	200 mL	80 mL	300 mL
		4.2	Fermentation time	t = 12 h	t = 48 h	t = 4 h	t = 12 h
	Cycle 5	5.1	Effluent/Feed volume	120 mL	-	80 mL	250 mL
		5.2	Fermentation time	t = 7h	-	t = 4 h	t = 4 h
	Cycle 6	6.1	Effluent/Feed volume	300 mL	-	200 mL	-
		6.2	Fermentation time	t = 7h	-	t = 16 h	-
	Cycle 7	7.1	Effluent/Feed volume	-	-	100 mL	-
		7.2	Fermentation time	-	-	t = 24 h	-
	Cycle 8	8.1	Effluent/Feed volume	-	-	10 mL	-
		8.2	Fermentation time	-	-	t = 24 h	-

**Table 4:** Results from *E. coli* A1:ldhA strain adaptation to the real biological feedstock.

Minimal Media M9	Feedstock F1	t (h)	OD_600_	Maltose (g/L)	Glucose (g/L)	AA (g/L)	LA (g/L)	Comments
80%	20%	0	0.165 ± 0.009	0.43 ± 0.01	0.66 ± 0.01	1.22 ± 0.02	13.68 ± 0.17	
		24	1.209 ± 0.012	0.10 ± 0.00	0.00 ± 0.00	0.82 ± 0.00	13.71 ± 0.04	
60%	40%	0	0.172 ± 0.003	0.98 ± 0.01	0.56 ± 0.00	1.81 ± 0.02	13.39 ± 0.09	
		24	1.035 ± 0.008	0.50 ± 0.01	0.04 ± 0.02	1.76 ± 0.02	13.27 ± 0.21	
40%	60%	0	0.179 ± 0.005	1.52 ± 0.05	0.44 ± 0.00	2.44 ± 0.06	13.16 ± 0.38	
		24	1.031 ± 0.011	0.47 ± 0.01	0.06 ± 0.00	2.66 ± 0.07	12.86 ± 0.32	
20%	80%	0	0.151 ± 0.001	2.09 ± 0.03	0.36 ± 0.00	2.98 ± 0.02	12.50 ± 0.10	
		24	0.876 ± 0.001	1.09 ± 0.14	0.04 ± 0.01	3.71 ± 0.01	12.25 ± 0.01	
		48	1.050 ± 0.009	0.73 ± 0.00	0.00 ± 0.01	4.10 ± 0.02	12.39 ± 0.06	
0%	100%	0	0.171 ± 0.003	2.64 ± 0.04	0.22 ± 0.01	3.65 ± 0.14	12.41 ± 0.42	Inoculated from 40% M9:60% biological feedstock
		24	0.855 ± 0.010	0.82 ± 0.05	0.00 ± 0.00	3.75 ± 0.04	12.43 ± 0.06	
		48	1.054 ± 0.012	0.60 ± 0.02	0.00 ± 0.00	3.00 ± 0.01	12.16 ± 0.01	
		24	0.880 ± 0.010	0.69 ± 0.01	0.00 ± 0.00	4.14 ± 0.04	12.49 ± 0.11	Inoculated from 20% M9:80% biological feedstock
		24	1.210 ± 0.015	0.60 ± 0.01	0.00 ± 0.00	4.03 ± 0.02	12.31 ± 0.01	Inoculated from 100% biological feedstock

**Table 5:** Calculated metrics for the comparative assessment of the MBR bio-purification process during semi-continuous operation (stage 2).

MBR Experiment	Second Stage Duration	Max |TMP| (mbar)	Max AA Consumption Rate (g/L/h)	Max Maltose Consumption Rate (g/L/h)	Net Recovery Rate (mL/h)	Max Maltose Removal (%)	Max AA Removal (%)
1	96	72	0.058	0.065	13.75	67.3	25.0
2	87	440	0.081	0.063	9.19	100.0	60.9
3	138	946	0.040	0.040	10.04	66.0	33.7
4	27	45	0.587	n.a.	44.29	100.0	100.0

## Data Availability

The data presented in this study are included in this article/Supplementary Materials; further inquiries can be directed to the corresponding author.

## Supplementary Materials

The following supporting information can be downloaded at: https://www.mdpi.com/article/10.3390/bioengineering11050412/s1, Figure S1: A photo of the nanofiltration (NF) unit used in this study; Figure S2: Permeability vs. permeate recovery during the NF test; Figure S3: NF permeate and initial feedstock; an NF membrane process step was applied on the real feedstock.
