# Supplementary material for: A Novel Bio-Purification Process Employing an Engineered E. coli Strain for Downstream Processing of Lactic Acid Solutions from the Fermentation of Agro-Industrial by-Products
Source: Bioengineering (Basel). 2024 Apr 23;11(5):412. doi: 10.3390/bioengineering11050412 (PMC11118208; doi:10.3390/bioengineering11050412)
Supplement: Supplementary file 1 [file bioengineering-11-00412-s001.zip › bioengineering-2942895-supplementary.pdf]

Article

A Novel Bio-Purification Process Employing an Engineered *E. coli* Strain for Downstream Processing of Lactic Acid Solutions from the Fermentation of Agro-Industrial by-Products

Alexandra Nastouli [1,2], Alexandra Moschona [1], Panagiotis A. Bizirtsakis [1], Joseph Sweeney [3], Irini Angelidaki [4], Michael Harasek [2], Anastasios J. Karabelas [1] and Sotiris I. Patsios [1,*]

[1] Laboratory of Natural Resources and Renewable Energies, Chemical Process & Energy Resources Institute (CPERI), Centre for Research and Technology-Hellas (CERTH), GR 57001 Thessaloniki, Greece; a.nastouli@certh.gr (A.N.); alexmoschona@certh.gr (A.M.); pbizirtsakis@certh.gr (P.A.B.); karabaj@certh.gr (A.J.K.)
[2] Institute of Chemical, Environmental and Bioscience Engineering, TU Wien, AU 1040 Vienna, Austria; michael.harasek@tuwien.ac.at
[3] School of Biosystems and Food Engineering, University College Dublin (UCD), D04 V1W8 Belfield, Dublin, Ireland; joseph.sweeney@ucd.ie
[4] Department of Chemical and Biochemical Engineering, Technical University of Denmark, DK-2800 Kongens Lyngby, Denmark; iria@kt.dtu.dk
* Correspondence: patsios@certh.gr; Tel.: +30-23-1049-8183

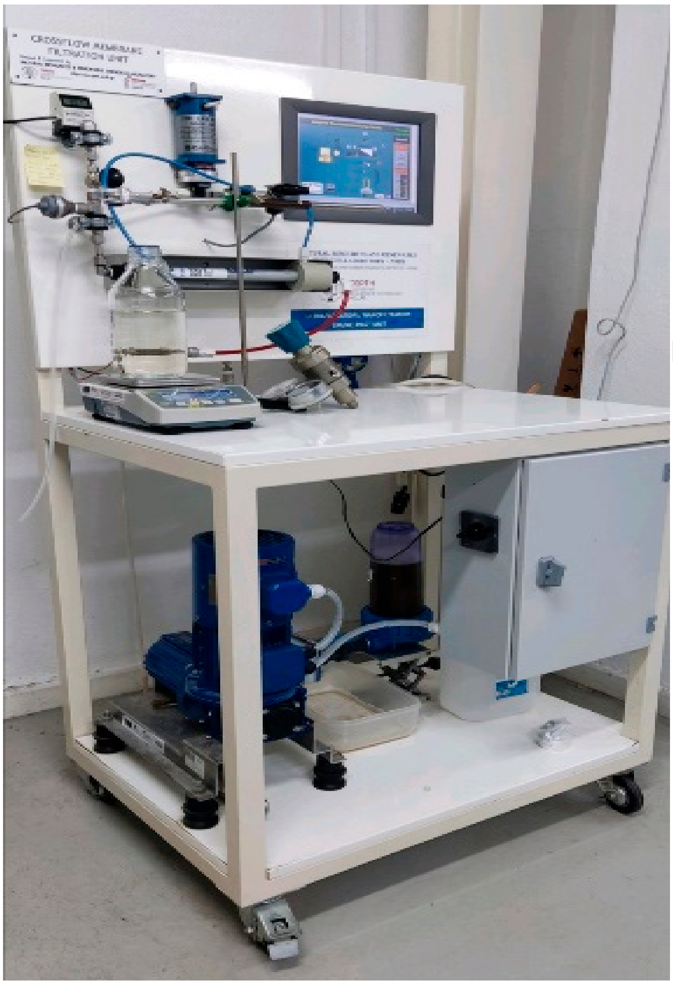

Figure S1. A photo of the nanofiltration (NF) unit used in this study.

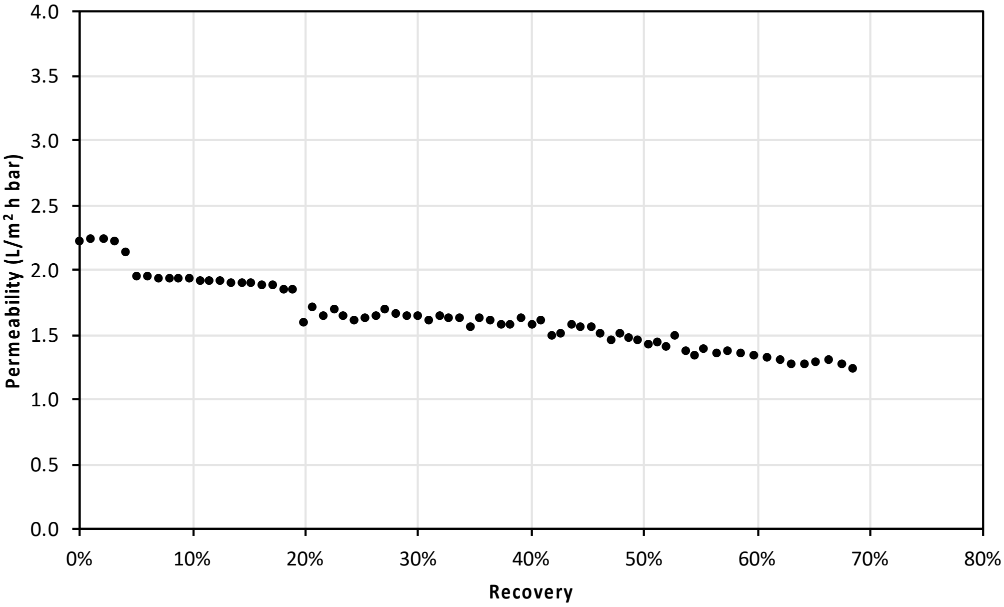

Figure S2. Permeability vs. permeate recovery during the NF test.

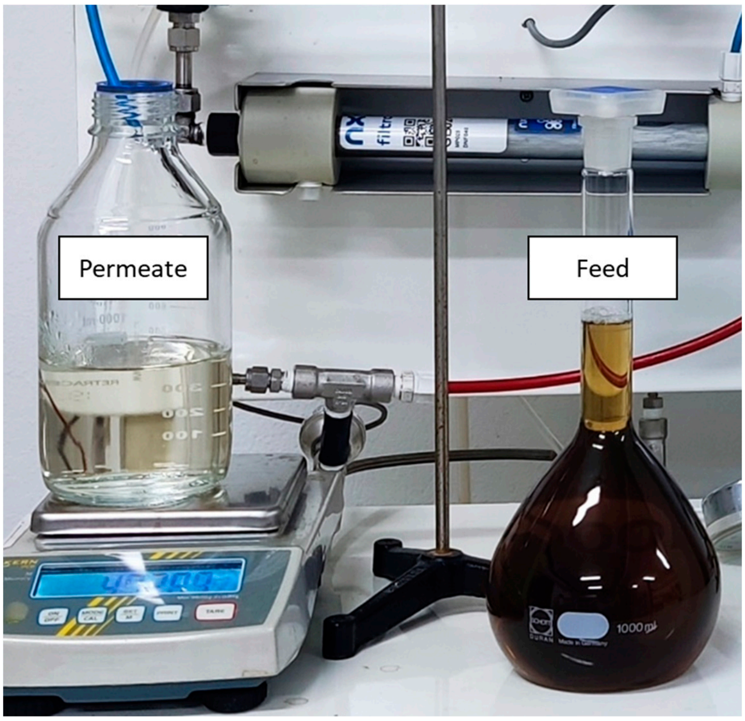

Figure S3. NF permeate and initial feedstock; an NF membrane process step was applied on the real feedstock.
